# Cancer derived peptide of vacuolar ATPase ‘a2’ isoform promotes neutrophil migration by autocrine secretion of IL-8

**DOI:** 10.1038/srep36865

**Published:** 2016-11-15

**Authors:** Safaa A. Ibrahim, Arpita Kulshrestha, Gajendra K. Katara, Magdy A. Amin, Kenneth D. Beaman

**Affiliations:** 1 Department of Microbiology and Immunology, Rosalind Franklin University of Medicine and Science, North Chicago, IL, USA; 2 Department of Microbiology and Immunology, Faculty of Pharmacy, Cairo University, Egypt

## Abstract

Neutrophils play significant regulatory roles within the tumor microenvironment by directly promoting tumor progression that leads to poor clinical outcomes. Identifying the tumor associated molecules that regulate neutrophil infiltration into tumors may provide new and specific therapeutic targets for cancer treatment. The a2-isoform of vacuolar ATPase (a2V) is uniquely and highly expressed on cancer cell plasma membrane. Cancer cells secrete a peptide from a2V (a2NTD) that promotes the pro-tumorigenic properties of neutrophils. This provides a2V the propensity to control neutrophil migration. Here, we report that the treatment of human neutrophils with recombinant a2NTD leads to neutrophil adherence and polarization. Moreover, a2NTD treatment activates surface adhesion receptors, as well as FAK and Src kinases that are essential regulators of the migration process in neutrophils. Functional analysis reveals that a2NTD can act as a chemo-attractant and promotes neutrophil migration. In addition, a2Neuɸ secrete high levels of IL-8 via NF-κB pathway activation. Confirmatory assays demonstrate that the promoted migration of a2Neuɸ was dependent on the autocrine secretion of IL-8 from a2Neuɸ. These findings demonstrate for the first time the direct regulatory role of cancer associated a2-isoform V-ATPase on neutrophil migration, suggesting a2V as a potential target for cancer therapy.

Leukocyte infiltration is a key feature of cancer-associated inflammation^1^. The increased frequency of tumor-infiltrating (associated) neutrophils (TAN) has been observed and was positively correlated with poor clinical outcomes in many tumor entities^1^,^2^,^3^. This clinical impact is dependent on the strong bidirectional crosstalk between neutrophils and tumor cells that lead to changes in neutrophils as well as the biology of tumor cells. These changes stimulate the pro-tumorigenic properties in TAN. Many of these TAN pro-tumorigenic features are shared with granulocyte-myeloid derived suppressor cells (G-MDSC), that are important in cancer establishment and progression^1^,^3^.

Important initiators that mediate the interaction between tumor cells and leukocytes are the vacuolar ATPases (V-ATPases); that are highly expressed on cancer cell surface^4^,^5^,^6^,^7^,^8^,^9^. V-ATPases are novel targets for anticancer therapeutics^4^,^5^. However V-ATPases are also important for various other physiological processes^10^. Thus, identifying selective targets of the V-ATPases is important to develop effective cancer therapy with minimized toxicity.

Neutrophils are the key effector cells in innate immunity and are the first responders to inflammatory stimuli^11^. Although neutrophils are short living cells, the continuous recruitment of neutrophils into tumors makes TAN capable of promoting tumor growth and metastasis^1^,^12^,^13^,^14^. This continuous migration of TAN is chiefly mediated by the secretion of the neutrophil chemo-attractant; IL-8 in the tumor microenvironment^14^,^15^. At the tumor site, IL-8 is secreted from tumor cells as well as inflammatory cells; however the driving factors that stimulate IL-8 secretion remain unclear. IL-8 binds to two different G protein coupled receptors on neutrophils; CXCR1 and CXCR2^11^. IL-8 binding activates these receptors and initiates a specific intracellular signaling cascade that induces neutrophil polarization and their rapid recruitment towards the tumor site^16^,^17^. IL-8 gene expression is up-regulated by the activation of the transcription factor NF-κB that is a key orchestrator of innate immunity and is activated in both tumor and inflammatory cells^17^,^18^. NF-κB consists of homo- or heterodimers of the Rel family proteins. The most common heterodimeric pair is p50/p65^19^, which is thought to be central to the regulation of numerous inflammatory response genes^18^.

The importance of neutrophils in cancer progression has triggered numerous efforts to therapeutically target these unique cells^3^,^16^,^20^. Current strategies in this area focus on the inhibition of either TAN recruitment or the pro-tumorigenic function of TAN^3^,^20^. Our previous studies have shown that specifically the a2-isoform of the ‘a’ subunit of V-ATPase (a2V) plays a significant role in the inflammation associated with cancer and pregnancy^8^,^21^,^22^. In cancer cells, a2V is highly expressed on the plasma membrane and the N-terminal domain of a2V (a2NTD) is secreted in microvesicles^6^,^8^,^9^,^21^. a2NTD stimulates M2 polarization of the macrophages, which behave as TAM and have a positive impact on tumor progression^1^,^6^,^8^. Our recent study showed that a2NTD promotes the pro-tumoral properties of neutrophils that stimulate the tumor cell invasion and angiogenesis^23^. The tumor signals that stimulate the neutrophil migration towards the tumor sites are not well defined. In our previous study, we showed that the increased a2NTD expression was associated with a significant increase in numbers of tumor infiltrating neutrophils in human invasive breast cancer^23^. Together, we hypothesized that a2NTD can play an important role in the neutrophil recruitment toward tumors.

In fact, we demonstrate here that a2NTD is directly involved in the enhancement of neutrophil migration. a2NTD treatment is sufficient to induce rapid neutrophil adherence, polarization as well as activation of important regulators of the migration process without prior stimulation. Importantly, a2NTD treatment activates the NF-κB pathway in neutrophils which leads to increased IL-8 secretion that is directly involved in the promoted a2NTD treated neutrophil (a2Neuɸ) migration. To our knowledge this is the first study demonstrating the involvement of tumor associated V-ATPases in regulating neutrophil migration by stimulating autocrine secretion of IL-8. These findings shed light on a2V and its soluble protein as potential new targets for cancer immunotherapy.

## Results

### a2NTD treatment stimulates neutrophil polarization and actin assembly

**Figure 1:**
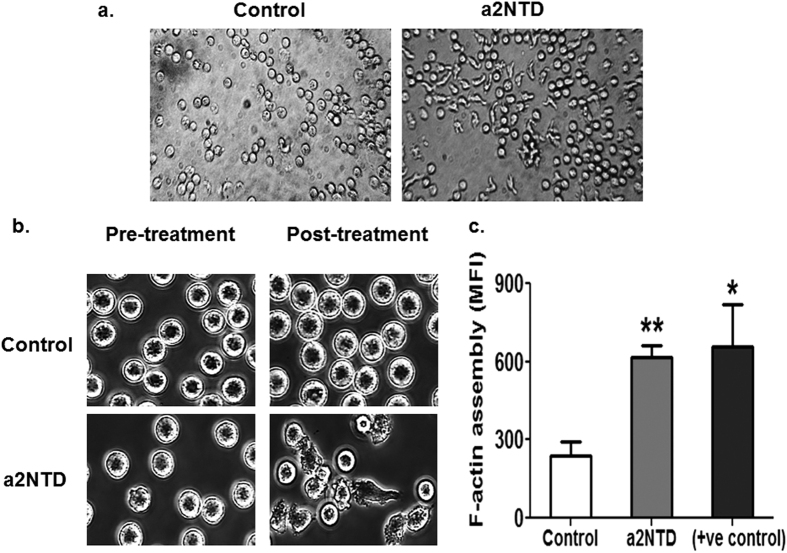
a2NTD treatment stimulates neutrophils polarization and F-actin assembly. (**a**) phase contrast images collected after one hour of a2NTD treatment under inverted light microscope (400x magnification). (**b**) Live cell imaging of neutrophils was performed before and after treatment with either PBS (vehicle control) or a2NTD using FV10i-LIV Laser Scanning Microscope (Olympus). Representative images collected after thirty minutes of treatment from two independent experiments are shown (600x magnification, 1.8x zooming). (**c**) F-actin assembly in neutrophils was quantified by flow cytometry using phalloidin AF488. Neutrophils were treated with PBS vehicle control, a2NTD or FMLP (positive control). Data were collected from three independent experiments done in duplicates and reported as mean fluorescence intensity (MFI) ± SEM. The statistical significance was compared with control neutrophils, *P < *0.05*, **P < *0.01*.

Neutrophils polarize into head and tail in order to migrate towards inflammation sites^24^. Here, a significant change in the neutrophil morphology was observed under the inverted light microscope, within one hour of treatment with recombinant a2NTD (Fig. 1a). In order to investigate the effect of a2NTD on the change of neutrophil morphology, we performed live cell imaging analysis of the neutrophils before and after a2NTD or vehicle treatment using live cell imaging confocal microscopy. Neutrophils began to adhere to the gelatin coated plate and formed extended lamellipodia; sheet-like protrusive structures and filopodia; finger-like protrusions and exhibited a polarized morphology within thirty minutes of a2NTD treatment (Fig. 1b, Supplementary Fig. S1 and S2). In the PBS treated neutrophils (vehicle control), this polarized phenotype was absent and the cells remained rounded (Supplementary Fig. S3). In the migration process, actin monomers assemble and form actin filaments (F-actin) that are crucial for lamellipodia and filopodia formation^25^. Flow cytometric analysis was performed to examine the effect of a2NTD treatment on F-actin formation in neutrophils using phalloidin AF-488; which binds to F-actin (Fig. 1c). a2Neuɸ showed significantly increased levels of F-actin, a 2.16 ± 0.16-fold increase, P < 0.01, relative to the control. These results showed that a2NTD treatment leads to increased actin assembly as well as the formation of extended filopodia in neutrophils suggesting that, a2NTD at the tumor site, can play an important role in neutrophil recruitment.

### Increased surface expression and activation of adhesion receptors in a2NTD treated neutrophils

**Figure 2:**
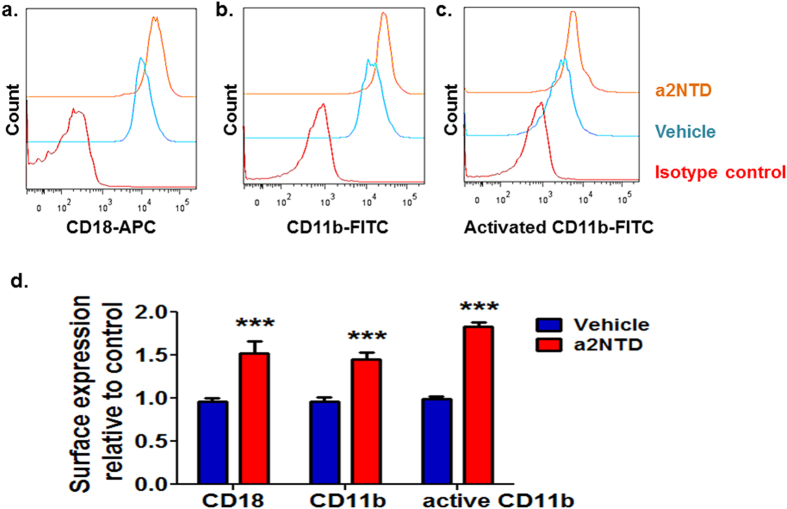
Increased surface expression and activation of adhesion receptors in a2NTD treated neutrophils. Flow cytometric analysis of the surface expression and the activation of CD11b/CD18 integrins. Neutrophils were treated with PBS (vehicle control) or a2NTD for one hour. Neutrophils were gated as CD15^+^/SSC^high^ population, and the surface expression of (**a**), CD18, (**b**), CD11b and (**c**), active CD11b were assessed. Representative histograms were presented from three different experiments each was done in duplicate. (**d**) The expression was presented as the mean fluorescence intensity relative to control ± SEM. The statistical significance was compared with vehicle control neutrophils, ***P < *0.001*.

Signaling via adhesion receptors of the β_2_ integrin family (CD11/CD18) plays an essential role in neutrophil recruitment and activation during inflammation. Mac-1 (CD11b/CD18, αMβ2) integrin is the most abundant β2 integrin on neutrophils^26^. Flow cytometric analysis of the surface expression of CD11b/CD18 revealed that a2Neuɸ expressed significantly elevated levels of CD11b and CD18 (45.6% ± 8.2 and 52% ± 13.8 percent increase in comparison to control, P < 0.001, respectively) (Fig. 2a,b,d). Importantly, the activated form of integrins activates the downstream signaling, which drives forward the migration process^27^. Therefore, we quantified the levels of the activated CD11b on the surface of the neutrophils after a2NTD treatment, using flow cytometry. a2NTD treatment significantly enhanced the surface expression of activated CD11b on neutrophils (83% ± 4.9 percent increase relative to control, P < 0.001) (Fig. 2c,d). Together, these data demonstrate the stimulatory effect of a2NTD on these adhesion receptors expression and activation, suggesting that a2NTD treatment can activate the downstream signaling that is involved in the migration process of neutrophils.

### Tyrosine kinases activation in neutrophils upon a2NTD treatment

**Figure 3:**
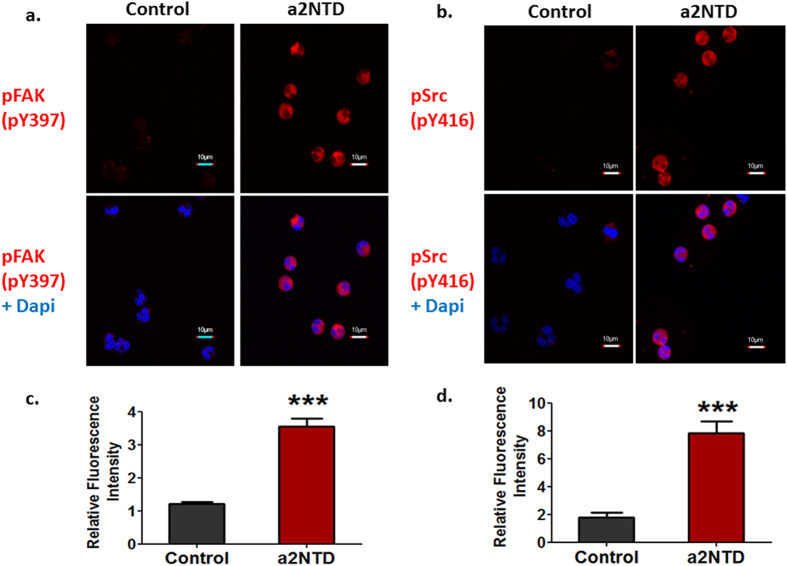
Tyrosine kinases activation in neutrophils upon a2NTD treatment. Immunofluorescent staining of (**a**), phosphorylated FAK (pY397) or (**b**), phosphorylated Src (pY416) was performed in neutrophils using confocal microscopy. Neutrophils treated with either PBS (vehicle control) or a2NTD were plated on poly Lysine coated 8 well chamber slides for thirty minutes at 37 °C CO_2_ incubator. Cells were fixed, permeabilized, and stained with anti-pFAK (pY397) or anti-pSrc (pY416) primary antibody, followed by secondary antibody AF594 (red), Dapi (blue). Representative images were presented from 3 different experiments. Original maginification 600x; scale bars; 10 μm. Quantification of the intensity of the immunofluorescent staining of (**c**), pFAK (pY397) or (**d**), pSrc (pY416) in neutrophils was performed using FV10i Fluoview Ver.3.0 software. Data are shown as relative average fluorescence intensity to control ± SEM. ***P < *0.001,* as compared with control neutrophils.

Integrin activation leads to activation of Focal adhesion kinase (FAK) by inducing autophosphorylation at Tyr397^28^. In order to investigate the activation status of FAK, freshly isolated neutrophils were plated on Lysine coated eight well chambered slide and treated with a2NTD or PBS for one hour. Immunofluorescent analysis was performed using specific antibodies against phospho-FAK (pY397) or total FAK. a2NTD treatment led to the activation of FAK by the enhancement of the phosphorylation at Tyr397 without changing the total FAK expression (Fig. 3a,c and Supplementary Fig. S4). In addition, the phosphorylation of FAK at Tyr397 activates Sarcoma-family kinases (Src kinases)^28^. Phosphorylation of Src kinases at Tyr416 leads to their activation^29^. Immunofluorescent analysis demonstrated that a2Neuɸ showed increased levels of the activated phosphorylated form of Src kinase (pY416) (Fig. 3b,d). These data show that the activation of these tyrosine kinases in neutrophils after a2NTD treatment can be a result of the integrin activation that leads to enhanced migration.

### a2NTD treatment promotes neutrophil migration

**Figure 4:**
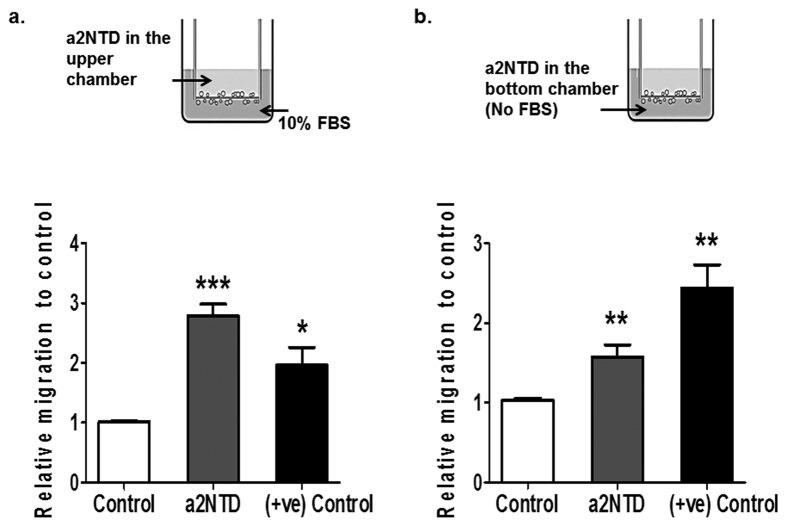
a2NTD treatment promotes neutrophil migration. The chemotactic activity of a2NTD was evaluated using fluorometric trans-well migration assay in two different procedures. (**a**) 4 × 10^5^ neutrophils were plated in the upper chamber in serum free media and stimulated with PBS (vehicle control), a2NTD or FMLP (+ve control), while 10% heat inactivated FBS complete media was the chemo-attractant in the bottom chamber. (**b**) modified trans-well migration assay was performed in serum free media. Neutrophils were plated in the upper chamber while PBS (vehicle control), a2NTD or FMLP (+ve control) were added to the bottom chamber. The migrated cells were quantified fluorometrically after three hours of incubation. Data were collected from 3 independent experiments done in triplicates. The statistical significance was compared with control neutrophils, *P < *0.05*, **P < *0.01*, ***P < *0.001.*

Since a2Neuɸ represented this polarized morphology and the activation of the adhesion receptors and regulators, we further investigated the functional ability of a2Neuɸ to migrate *in vitro* by transwell migration assay. Neutrophils were stimulated with a2NTD and allowed to migrate towards a 10 percent heat inactivated FBS supplemented media in the bottom chamber for three hours. The migrated neutrophils were quantified using a fluorometric assay. a2NTD treatment led to increased migration of neutrophils with a 2.8 ± 0.19-fold increase as compared with control neutrophils, P < 0.001, (Fig. 4a). Moreover, a modified transwell migration assay was performed to investigate the ability of a2NTD a lone to promote neutrophil migration; where a2NTD was added to serum free media in the bottom chamber without the addition of any other chemo-attractant (Fig. 4b). Neutrophils were loaded to the upper chamber in a serum free media and allowed to migrate to the bottom chamber for three hours. a2NTD promotes neutrophil migration significantly towards the bottom chamber by 1.57 ± 0.15-fold increase, P < 0.01, relative to control. Also, we performed the transwell migration assay using tumor conditioned media (TCM) collected from MDA MB-231; highly invasive breast cancer cell line, that highly expresses a2V and secretes a2NTD in the microvesicles^8^,^23^ to assess the ability of TCM (in the bottom chamber) to attract neutrophils. The TCM showed similar effect as a2NTD that it increases neutrophil migration by 2.95 ± 0.5, fold increase, P < 0.05, relative to control media (Supplementary Fig. S5a). These data confirmed that a2NTD increased the migration ability of neutrophils and suggest that a2NTD can promote neutrophil migration towards the tumor site that can lead to tumor progression.

### a2NTD treatment leads to NF-κB pathway activation in neutrophils that induces IL-8 secretion

**Figure 5:**
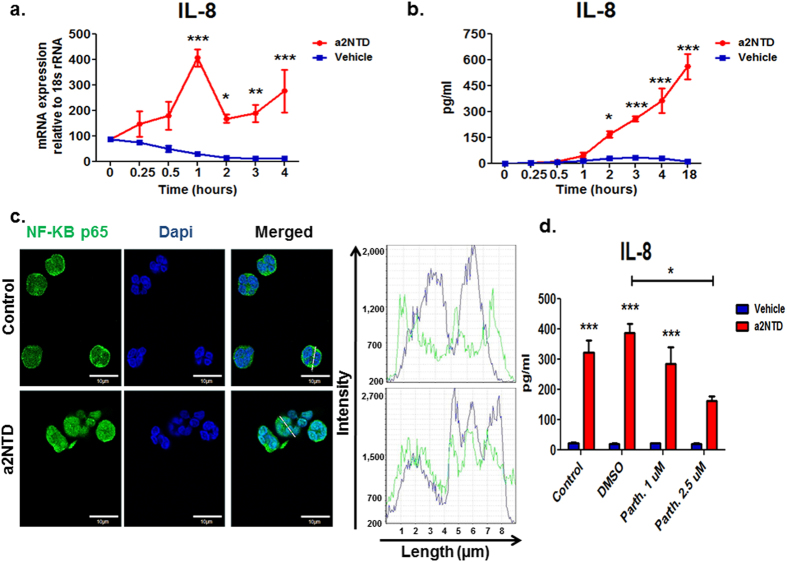
a2NTD treatment leads to NF-κB pathway activation in neutrophils that induces IL-8 secretion. (**a**) Quantitative real time-PCR was performed to assess the mRNA expression of IL-8 in a2NTD or PBS (vehicle control) treated neutrophils after different time point of treatment. Data were reported as mRNA expression relative to 18s rRNA ± SEM from at least three different experiments each was done in duplicate. *P < *0.05*, **P < *0.01*, ***P < *0.001* as compared with vehicle treated neutrophils. (**b**) Quantitative analysis of IL-8 secreted levels was assessed using Luminex assay. IL-8 protein levels were determined in the supernatant collected from a2NTD or vehicle treated neutrophils after different time point of treatment. Results presented as mean ± SEM from four different experiments. *P < *0.05*, ***P < *0.001* as compared with vehicle treated neutrophils. (**c**) Immunofluorescent analysis of NF-κB p65 expression was performed after thirty minutes treatment of neutrophils with either a2NTD or PBS (vehicle control). Cells were examined by confocal microscopy, NF-κB p65 (green), Nucleus (blue) and co-localization of NF- κB p65 with the nucleus represents NF-κB p65 activation. Histograms represent the Intensity of the staining of the white highlighted region crossing the cells; blue represents Dapi staining intensity and green represents NF- κB p65 staining intensity. Original maginification 600x; scale bars; 10 μm. Representive images are presented from 3 different experiments. (**d**) Effect of Parthenolide (NF-κB inhibitor) on IL-8 secreted levels from neutrophils after four hours of treatment was performed using Luminex assay. Results presented as mean ± SEM from four different experiments. *P < *0.05*, ***P < *0.001*, as compared with control neutrophils.

IL-8 is a chemo-attractant, secreted by various types of cells in the tumor microenvironment including tumor associated neutrophils^16^,^17^,^30^. To investigate the effect of a2NTD on the kinetics of IL-8 expression in neutrophils, we quantified the IL-8 gene expression in a2Neuɸ at different time points of treatment using quantitative RT-PCR. a2NTD treatment led to increased gene expression of IL-8 starting from fifteen minutes. The IL-8 expression increases significantly after one hour of treatment to reach 17.2-fold increase relative to control, P < 0.001, (Fig. 5a). Interestingly, IL-8 mRNA expression decreased after two hours of treatment to a 12.4-fold increase and then a time-dependent increase was observed to a 29-fold increase after four hours of a2NTD treatment. Similarly, neutrophil IL-8 secretion levels increased starting from thirty minutes of a2NTD treatment and continued increasing up to eighteen hours of treatment (561.67 pg/ml ± 73.2, P < 0.001) as compared with control neutrophils (10.94 pg/ml ± 2.59) (Fig. 5b). Also we confirmed that tumor conditioned media (TCM) collected from MDA MB-231 exerts similar effects as a2NTD on IL-8 mRNA expression and secretion. Our results showed that the treatment of neutrophils with TCM for four hours led to increase the IL-8 mRNA gene expression by 30.06 ± 21.9 fold increase, P < 0.05, relative to control neutrophils (Supplementary Fig. S5b). Similarly, neutrophil IL-8 secretion levels elevated significantly after eighteen hours of treatment with TCM (2848.6 ± 306.3 pg/ml, P < 0.05) as compared with IL-8 levels present in TCM (1573.7 ± 132.1 pg/ml) (Supplementary Fig. S5c).

The NF-κB pathway had a central role in regulating the transcription of cytokines, adhesion molecules, and other mediators^31^. Expression of IL-8 is primarily regulated by NF-κB-mediated transcriptional activity^17^,^18^. Immunofluorescent analysis revealed that NF-κB p65 translocates into the nucleus upon a2NTD treatment of the neutrophils, which indicates the activation of NF-κB pathway (Fig. 5c). This activation was observed as early as thirty minutes after a2NTD treatment. These data demonstrate that a2NTD treatment upregulated IL-8 expression can be as a result of the activation of the NF-κB pathway.

In order to confirm this observation, parthenolide; an NF-κB inhibitor was used and the levels of the secreted IL-8 were quantified by the Luminex assay after four hours of treatment (Fig. 5d). Inhibiting NF-κB hampered the secretion of IL-8 in the a2Neuɸ (161.74 pg/ml ± 16.7, P < 0.05) as compared to a2NTD treated control neutrophils (389.13 pg/ml ± 86.7). These data confirm the regulatory role of a2NTD on stimulating IL-8 secretion by neutrophils via NF-κB pathway activation.

### a2NTD treated neutrophils exhibit enhanced migration through IL-8/CXCR1-2 axis

**Figure 6:**
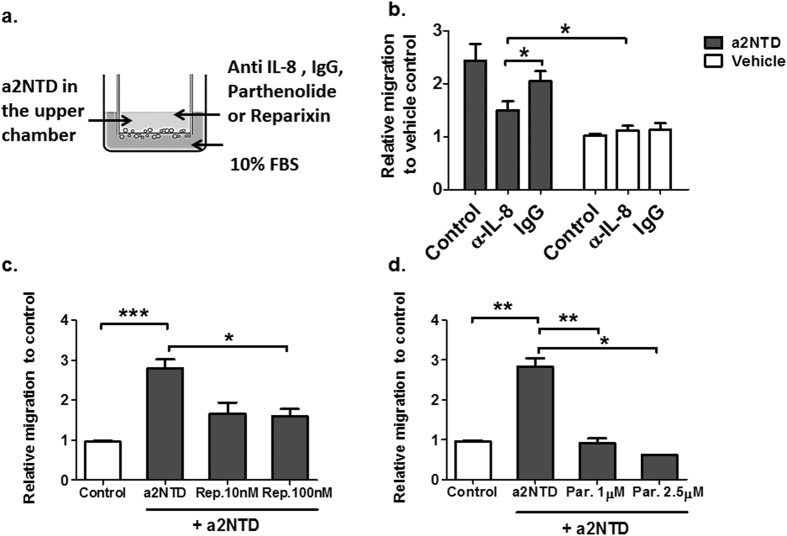
a2NTD treated neutrophils showed increased migration through autocrine secretion of IL-8. (**a**) trans-well migration assays were performed by plating 4 × 10^5^ neutrophils into the upper insert and treated with (**b**) neutralizing anti-IL-8 or Isotype IgG or (**c**) Reparixin; CXCR1-2 inhibitor or (**d**) Parthenolide; NF-κB inhibitor for fifteen minutes followed by PBS or a2NTD treatment for three hours. 10% heat inactivated FBS was used as a chemo-attractant in the lower chamber. The migrated cells were quantified using fluorometric assay. Data were collected from 3 independent experiments done in triplicates. The statistical significance was compared with vehicle treated neutrophils, *P *<* *0.05*, **P < *0.01*, ***P < *0.001.*

To determine whether the secreted IL-8 from a2Neuɸ is responsible for neutrophil enhanced migration, a transwell migration assay was performed as described in the materials and methods section using a neutralizing antibody against IL-8 (Fig. 6a,b). The addition of anti-IL-8 hampered the migration of a2Neuɸ from 2.45 ± 0.3 to 1.49 ± 0.16-fold increase relative to vehicle treated control neutrophils, P < 0.05. However, there was no change in the migration status of the PBS treated neutrophils upon addition of anti-IL-8, confirming that the stimulated migration of a2Neuɸ was due to the increased secretion of IL-8.

IL-8 binds to and signals through two G-protein-coupled receptors, IL-8RA (CXCR1) and IL-8RB (CXCR2), which are present on neutrophils^17^. In order to decipher whether or not the enhanced migration of a2Neuɸ is due to IL-8 binding to CXCR1-2, neutrophils were treated for fifteen minutes with Reparixin, which is an IL-8 allosteric antagonist that inhibits the activation of CXCR-1 and CXCR-2 chemokine receptors^20^. Neutrophils were then challenged with a2NTD and allowed to migrate for three hours (Fig. 6c). Using Reparixin significantly abrogated a2NTD induced migration of neutrophils from 2.54 ± 0.14 to 1.6 ± 0.17-fold increase relative to vehicle treated neutrophils, P < 0.05. Furthermore, Parthenolide; NF-κB activation inhibitor, negated the stimulatory effect of a2NTD on neutrophil migration (Fig. 6d). These results confirm that a2NTD treatment activates the NF-κB pathway in neutrophils, which leads to the upregulation of IL-8 expression and secretion that induces neutrophil migration. It also demonstrates that a2Neuɸ increased migration was primarily due to the autocrine secretion of IL-8 which binds to CXCR1-2. Together, these data suggest that tumor associated a2V-ATPase plays an important role in neutrophil recruitment to the tumor site, by the action of a2NTD.

## Discussion

Neutrophils make up a significant portion of the inflammatory cell infiltrate found in a wide variety of human cancers as well as murine cancer models^2^,^16^. The importance of neutrophils in cancer biology is well established and they are considered to be a potential new target for cancer immunotherapy^1^,^16^,^20^. Thus, identifying the factors that regulate neutrophil migration to the tumor site is important for the development of targeted therapy. Here we show a novel mode of regulating neutrophil migration through the action of tumor associated V-ATPases. We present that a cleaved peptide from a2V-ATPases; a2NTD promotes neutrophil migration. We further demonstrate that this induced migration of a2Neuɸ is associated with increased expression and activation of neutrophil adhesion receptors and migration regulatory proteins. The mechanism of this promoted migration involves the activation of NF-κB pathway in neutrophils and substantial secretion of IL-8 which acts in an autocrine manner on neutrophils. These findings provide evidence to a novel regulatory role that a2V play in the tumor microenvironment, by its signaling peptide, a2NTD.

Neutrophils are the first responders to inflammatory stimuli^11^. Several clinical studies have pointed out that neutrophil accumulation in neoplasms confers a poor prognosis^1^,^2^. For instance, the presence of TAN in renal cell carcinoma correlates with increased mortality^32^. Also, the increased levels of TAN in patients with bronchio-alveolar carcinoma were significantly associated with poor outcomes^33^. Similarly, TAN is associated with the aggressive types of pancreatic neoplasms^34^. Notably, IL-8 levels in tumors have been associated with neutrophil accumulation and reduced survival^35^. On the other hand, the anti-cancer effect of neutrophils also has been reported in some cases that alter the tumor microenvironment, for instance, in TGFb1 signaling blockade^36^, in circumstances that shift the chronic inflammatory state toward an acute inflammatory response around a tumor^37^, In engineered tumors^38^, and in early-stage human lung cancer^39^. Also both pro- and antitumoral properties were observed simultaneously in a study by Zelvyte *et al.,* in which PMN-derived factors reduced lung cancer cell proliferation *in vitro*, but increased cell invasiveness^40^.

V-ATPases; multisubunit proton pumps are involved in cancer initiation as well as progression^5^,^41^. In contrast to normal cells, a2V is highly expressed on the surface of the cancer cells and a2NTD is secreted in microvesicles^1^,^6^,^8^,^9^,^21^,^23^. In addition to the direct role of a2V in tumor progression by the regulation of pH and invasion, a2V also has immunomodulatory roles in the tumor microenvironment via the released peptide a2NTD^6^,^7^,^42^. Our previous *in vitro* and *in vivo* models have provided evidence that tumor associated a2NTD regulates myeloid cells in a way that promotes tumor growth, angiogenesis and invasiveness^6^,^8^,^21^,^23^. Also we have previously shown by immunohistochemical analysis in invasive breast cancer tissues that increased neutrophil recruitment and blood vessel density were correlated with increased a2NTD levels^23^. In addition, functional characterization of a2Neuɸ revealed that a2Neuɸ derived products induce *in vitro* angiogenesis as well as increase the invasiveness of breast cancer cells and had no cytotoxic effect on breast cancer cell proliferation^23^. Here we investigated the role of a2NTD on neutrophil migration.

Neutrophil extravasation is a multistep process, including rolling, activation, adhesion and transendothelial migration^43^. Circulating naive neutrophils are apolar, but upon stimulation by chemo-attractants, they rapidly polarize in a manner that is represented by formation of lamellar-type F actin at the leading edge^44^. Similarly, in the present study, we showed that a2NTD treatment led to a dramatic change in neutrophil morphology in culture without any prior stimulation. Live-cell imaging analysis confirmed that a2NTD treatment stimulated neutrophils to rapidly adhere, polarize and to form extended lamellipodia and filopodia at the leading edge of the cells. These protrusions are actin based structures in which, actin monomers polymerize to form F-actin^25^. In Fact, we showed that increased levels of F-actin were formed in a2Neuɸ, confirming that a2NTD treatment led to the formation of these protrusion structures that drive increased migration of the neutrophils.

Other important players in the migration process are the members of the adhesion receptors of β_2_ integrin family that are known to be essential for neutrophil adhesion^45^. In neutrophils, β_2_ integrins mediate signaling predominantly via Mac-1 (CD11b/CD18, αMβ2) integrin^26^. Data from adhesion receptor analysis showed that the induced alterations in the a2Neuɸ phenotype were associated with increased surface expression of CD11b/CD18 integrin as well as increased levels of the activated form of these integrins. In agreement with our results, Wu *et al* reported that secreted factors from breast cancer cell lines stimulate neutrophil Mac-1 surface expression and stimulate neutrophils migration^46^,^47^.

In motile cells, protein tyrosine kinases such as the Focal adhesion kinases (FAK) and Src kinases, regulate the formation and remodeling of focal adhesions that are implicated in cell locomotion^48^. Focal adhesion kinase (FAK) is a widely expressed cytoplasmic protein tyrosine kinase involved in integrin-mediated signal transduction^48^. It is well known that integrin activation leads to the activation of FAK by inducing autophosphorylation at Tyr397^28^,^48^. Similarly, we showed that a2NTD activates FAK by inducing the Tyr397 phosphorylation of FAK in neutrophils, suggesting that Mac-1 activation leads to FAK activation. Also, phosphorylation of FAK at Tyr397; which is a binding site of Src Kinases, leads to activation of Src kinases^28^. The phosphorylation at Tyr416 presents in the activation loop of the Src kinase domain up-regulates enzyme activity^29^. Here we show that the phosphorylation of Src kinase at Tyr416 is associated with FAK activation in a2Neuɸ. Together we postulate that a2NTD treatment induces the activation of Mac-1 integrin in neutrophils that leads to the activation of the downstream tyrosine kinases that stimulate neutrophil migration.

Tumor cells secrete chemo-attractants to recruit leucocytes to the tumor site in order to create suitable microenvironment for tumor growth and metastasis^13^,^14^,^15^,^16^,^17^,^30^. We showed by *in vitro* functional analysis that a2NTD promotes neutrophil migration towards the chemo-attractant. Importantly, in a modified transwell migration assay; neutrophils migrated towards a2NTD, suggesting that a2NTD can act as a chemo-attractant of neutrophils. Together these data demonstrate that a2NTD in the tumor site can play an important role in the recruitment of neutrophils; that are known to promote tumor progression.

Inflammation is a critical component of tumor progression^49^. Abundant production of pro-inflammatory cytokines and chemokines in the tumor microenvironment can lead to a level of inflammation that potentiates angiogenesis, thus favoring cancer growth^14^,^49^. In the tumor microenvironment, IL-8 is secreted from different types of cells including tumor cells and immune cells. IL-8 is a potent neutrophil chemo-attractant and also possesses angiogenic activity that is associated with tumor angiogenesis and metastasis^17^. The factors regulating IL-8 secretion in tumor sites remain unclear^16^. Neutrophils produce cytokines and chemokines, which can impact inflammatory cell recruitment, thus altering the immune response towards tumor^1^,^16^,^30^. *In vitro* and *in vivo* studies reported that a2NTD regulates the gene expression and the secretion of various inflammatory mediators from myeloid cells that modulate the tumor progression^6^,^8^,^9^,^23^. Our results show that a2NTD treatment stimulates IL-8 gene expression as well as IL-8 secretion in neutrophils in a time dependent manner. IL-8 expression is primarily regulated by the NF- κB pathway^17^,^18^. The NF-κB complex is sequestered in the cytoplasm by inhibitory I-kB proteins. Upon cellular activation, the I-kB proteins degrade and this allows the NF-κB complex to translocate to the nucleus and activate gene transcription^18^,^31^. This is consistent with our findings that the increased IL-8 expression is associated with the nuclear translocation of NF-κB p65 that indicates its activation. Because of the DNA-binding sites for NF-κB are present in the I-kB-α promoter; activation of NF-κB is followed by increased expression of I-kBα in a regulatory loop aimed at normalization of NF-κB activation^31^. This can explain the slight decrease of IL-8 gene expression in a2Neuɸ after two hours of treatment or it may be due to other intervening pathways. In addition, inhibiting NF-κB activation resulted in a significant decrease in IL-8 secretion from a2Neuɸ. Together, these data suggest a new pathway of IL-8 production in tumor microenvironment, as a result of the interaction between tumor cells and neutrophils.

Neutrophils express chemokine receptors CXCR-1 and CXCR-2; that bind to IL-8 and stimulate neutrophil migration^11^. We demonstrate that the induced migration of a2Neuɸ is hampered significantly by anti-IL-8 neutralizing monoclonal antibody as well as by reparixin; a CXCR-1 and CXCR-2 inhibitor that is used in phase 2 clinical trial for Triple negative breast cancer treatment^50^. These data indicate that the increased migration of a2Neuɸ is controlled primarily by autocrine secretion of IL-8. Nevertheless, the fact that anti-IL-8 neutralizing monoclonal antibody and reparixin did not fully block a2Neuɸ promoted migration; however NF-κB inhibitors negated the a2NTD effect on neutrophil migration. These data indicate that other soluble factors secreted by a2Neuɸ can be involved in neutrophil induced migration. Also, we have shown previously that a2NTD stimulates neutrophils to secrete VEGF that is known to be a chemo-attractant, so it might play a part in the migration^23^. Further studies into the other mechanisms involved in a2NTD induced neutrophil migration are warranted to clarify our understanding of the link between a2V and cancer related inflammation.

**Figure 7:**
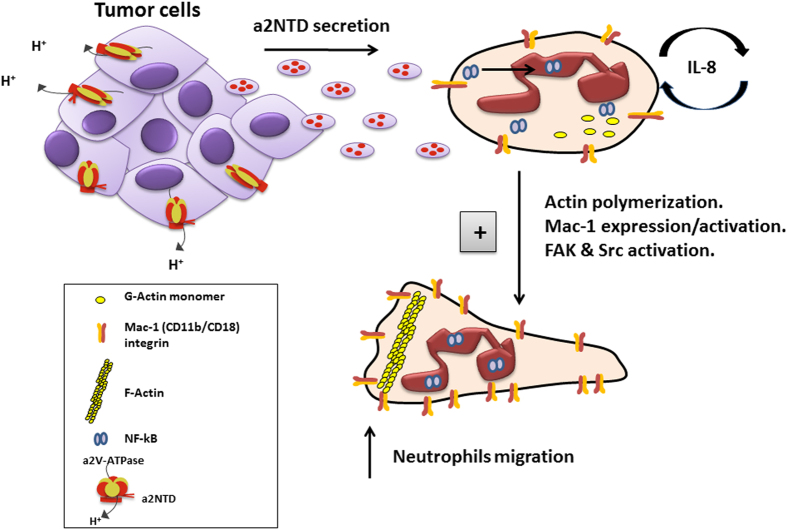
Schematic diagram depicting the proposed mechanism of the neutrophil induced migration by the effect of a2NTD. Cancer cells express a2V on the surface and secrete a2NTD in the released microvesicles. a2NTD activates NF-κB pathway in neutrophils that drives the synthesis and secretion of increased levels of IL-8 by neutrophils. Secreted IL-8 acts in an autocrine manner by binding to CXCR-1 and CXCR-2 on neutrophil. These are associated with actin polymerization, increased Mac-1expression and activation as well as FAK and Src activation. Together a2NTD treatment leads to promoted neutrophil migration suggesting a2NTD as a potential neutrophil chemo-attractant in tumor site.

In summary, this study demonstrates new insight to the link between tumor associated V-ATPase and innate immunity. We provide evidence that a2V soluble peptide; a2NTD activates the NF-κB pathway in neutrophils, which leads to an increased expression and secretion of IL-8 that stimulates neutrophil migration in an autocrine manner (Fig. 7). Inhibition of either neutrophil trafficking into tumors or their activation is a straightforward new approach to target neutrophils in cancer. This study shows how tumor associated a2-isoform V-ATPase can induce neutrophil migration by stimulating autocrine secretion of IL-8, suggesting a mechanism for the creation of a level of inflammation that favors cancer growth. Together these findings, along with our previous work that show the important role of the tumor associated a2V in promoting the pro-tumorigenic properties of neutrophils demonstrate that a2V and its cleaved peptide a2NTD can be a potential target for cancer immunotherapy.

## Materials and Methods

### Isolation of primary neutrophils from human blood

These studies were approved by the Rosalind Franklin University of Medicine and Science Institutional Review Board. After informed consent was obtained in accordance with the Declaration of Helsinki, peripheral blood was collected from healthy adult volunteers into sodium heparin vacutainers (Thermo Fisher Scientific). All experiments were performed in accordance with the guidelines of Rosalind Franklin University of Medicine and Science Environmental Health and Safety (EHS). Human neutrophils were isolated as described previously^23^. In brief, neutrophils were purified by dextran sedimentation and density-gradient centrifugation with Ficoll-Paque PLUS (Thermo Fisher Scientific) under endotoxin-free conditions. Contaminating red blood cells were removed by hypotonic lysis. The neutrophil cell pellet was re-suspended in MEM complete media (Invitrogen) containing 10% heat inactivated fetal bovine serum (FBS), penicillin (100 U/ml), streptomycin sulfate (100 mg/ml), and 2.0 mM glutamine in a cell density of 1 × 10^6^ cell/ml. Neutrophils purity was 97–99% (CD15/high side scatter) with 99% viability as determined by flowcytometry (AnnexinV/7-AAD^−/−^) and trypan blue exclusion. Neutrophils present in serum free media were recommended for some experiments. For that purpose neutrophils were washed two times with MEM serum free media and then suspended in the same media. Neutrophils were incubated at 37 °C in a humidified atmosphere containing 5% (v/v) CO_2_.

### Recombinant a2NTD

a2NTD was expressed and purified from *Escherichia coli* and subjected to endotoxin removal column chromatography (Proteome Resources, Aurora, CO) as previously described^8^,^9^.

### Live cell Imaging

Time-lapse imaging was performed using FV10i-LIV Laser Scanning Microscope (Olympus). 2.5 × 10^5^ Neutrophils were plated on Nunc™ glass bottom dishes (Thermo Fisher Scientific) coated with 0.2% gelatin. Imaging of the neutrophils was acquired before and after treatment with 1 μg/ml of a2NTD or PBS (a2NTD vehicle control). Images and videos were analyzed by FV10i Fluoview Ver.3.0 software.

### F-actin assembly assay

The quantification of F-actin in neutrophils was performed by FACs scan flow cytometry as described previously^51^. Briefly, the PBS (vehicle control), 500 ng/ml a2NTD or 100 nM FMLP (+ve control) treated neutrophils in serum free media were fixed, permeabilized using BD Cytofix/Cytoperm (BD Biosciences) and stained with 2 × 10^−8^ M Alexa Fluor^®^ 488 Phalloidin (Thermo Fisher Scientific) at room temperature for twenty minutes, and washed with PBS. Neutrophils were gated using high side scatter versus forward scatter. The results were expressed as mean fluorescence intensity (MFI).

### Surface expression of adhesion molecules (CD11b/CD18)

The expression of CD11b, CD18 and active form of CD11b on the surface of neutrophils was quantified by LSR II FACs scan flow cytometer. 1 × 10^6^ Neutrophils were treated with PBS (vehicle control) or a2NTD for one hour at 37 °C, in CO_2_ incubator. Direct immunofluorescence was performed using Pacific blue anti human CD15, FITC anti human CD11b, APC anti human CD18, FITC anti active CD11b or respective isotype controls (Biolgend) as described previously^46^. Neutrophils were gated as CD15^+^/SSC^high^. Measurements are presented as relative fluorescence index (RFI) by dividing the fluorescence value of the a2NTD treated groups by that of the control groups.

### RNA isolation and real time PCR

After treating neutrophils with PBS (vehicle control) or recombinant a2NTD or TCM for indicated time points incubation at 37 °C, 5% CO_2_ incubator, total RNA was isolated using RNeasy micro kit (Qiagen) and single-stranded cDNA was synthesized using QuantiTect reverse transcription kit (Qiagen). Qantitative real-time PCR (Q RT-PCR) was performed, using cDNA specific FAM-MGB–labeled Taqman primer sets (Applied Biosystems) for IL-8 gene and VIC-MGB labeled *18s rRNA* was used as endogenous control.

### Immunofluorescence analysis

1 × 10^5^ freshly isolated neutrophils suspended in serum free MEM media were treated with 500 ng/ml a2NTD or PBS (vehicle control) then plated on poly-L-lysine coated 8 well chamber slides. After thirty minutes incubation at 37 °C, CO_2_ incubator, cells were fixed with 4% paraformaldehyde, permeabilized by alcohol/acetone, blocked and stained with anti-human FAK (BD Biosciences), anti-human FAK (PY397) (BD Biosciences) or anti-human Src (PY416) (Cell Signaling) for one hour at room temperature. Alexa Fluor^®^ 594-conjugated donkey anti-mouse or Alexa Fluor^®^ 594-conjugated goat anti-rabbit secondary antibodies (1:200 dilution) (Invitrogen) was used. For the assessment of NF-κB p65 activation, neutrophils were treated as mentioned earlier and incubated for thirty minutes at 37 °C, CO_2_ incubator. Cells were fixed, cytospun on glass slides, permeabilized, blocked and stained with anti-human NF-κB p65 (Abcam). Alexa Fluor^®^ 488-conjugated goat anti-rabbit secondary antibodies (1:200 dilution) (Invitrogen) was used. The cells were prepared for viewing using ProLong^®^ Gold (Invitrogen) mounting medium containing DAPI. Stained cell lines were imaged by an Olympus Fluoview FV10i confocal microscope and analyzed by FV10i Fluoview Ver.3.0 software.

### IL-8 bioassay

The secretion of IL-8 was analyzed by Milliplex map kit (Millipore) in the supernatant of neutrophils (1 × 10^6^ cell/ml) collected after indicated time point of incubation, and assayed on a MAGPIX instrument (Millipore) as per the instructions provided by manufacture. Equal volumes from cell supernatant were used for the assay. The assay was performed at least for four independent experiments.

### Breast cancer cell conditioned medium

MDA-MB-231 human breast cancer cells were plated in MEM (Invitrogen) supplemented with 10% FBS (Life Technologies) at 37 °C, 5% CO_2_. Once cells were 60% to 70% confluent, the medium was removed, cells were washed twice with PBS, and fresh complete MEM media was added for 24 hours. After 24 hours, the medium was collected; centrifuged, filtered and conditioned medium was transferred to a new tube. Aliquots of the conditioned medium were stored at −20 °C.

### *In vitro* transwell migration assay

The transwell migration assay was conducted using the CytoSelect 24-well cell migration assay kit (Cell Biolabs Inc). Briefly, neutrophils (4 × 10^5^ cells/200 μl serum-free MEM media) were added to the upper chambers (3 μm pore size). 600 μl of MEM supplemented with 10% heat-inactivated FBS that act as a chemo-attractant, was added to the lower chamber. 500 ng/ml a2NTD, 100 nM FMLP (+ve control) or their respective control PBS or DMSO were added to the upper chamber. In the experiments using inhibitors; neutrophils were pretreated with Parthenolide (Millipore), Reparixin (Cayman chemical company), 10 μg/ml neutralizing (NA/LE) mouse anti human IL-8 antibody or 10 ug/ml Mouse IgG (BD Pharmingen) for fifteen minutes prior the addition of a2NTD. After three hour of incubation at 37 °C, CO_2_ incubator. Migrated neutrophils were collected from the lower surface of the membrane using detaching buffer provided with the kit. Also the suspended migrated neutrophils in the lower chamber were collected by centrifuging the media present in the lower chamber. Then migrated cells were quantified by fluorometric assay using ELISA plate reader according to the manufacturer’s instructions. A modified transwell migration assay was performed by adding serum free media containing a2NTD, FMLP or their respective controls to the bottom chamber. 4 × 10^5^ neutrophils/200 μl serum-free MEM media) were added to the upper chambers and allowed to migrate for three hours.

### Statistical analysis

Statistical analysis was performed with Mann–Whitney test or paired two-tailed Student’s *t-*test using Graph Pad Prism 5 (GraphPad Software, Inc., San Diego, CA, USA). Data are expressed as mean ± SEM. *P* value less than 0.05 was considered statistically significant different.

## Additional Information

**How to cite this article**: Ibrahim, S. A. *et al.* Cancer derived peptide of vacuolar ATPase ‘a2’ isoform promotes neutrophil migration by autocrine secretion of IL-8. *Sci. Rep.* **6**, 36865; doi: 10.1038/srep36865 (2016).

**Publisher’s note:** Springer Nature remains neutral with regard to jurisdictional claims in published maps and institutional affiliations.

## Supplementary Material

Supplementary Video S1

Supplementary Video S2

Supplementary Video S3

Supplementary Information
