# Supplementary material for: Cancer derived peptide of vacuolar ATPase ‘a2’ isoform promotes neutrophil migration by autocrine secretion of IL-8
Source: Sci Rep. 2016 Nov 15;6:36865. doi: 10.1038/srep36865 (PMC5109272; doi:10.1038/srep36865)
Supplement: Supplementary Information [file srep36865-s4.pdf]

Title: Cancer derived peptide of vacuolar ATPase 'a2' isoform promotes neutrophil migration by autocrine secretion of IL-8

Authors: Safaa A. Ibrahim[1,2], Arpita Kulshrestha[1], Gajendra K. Katara[1], Magdy A. Amin[2] and Kenneth D. Beaman[1]*

Supplementary Data:

Supplementary Figure legends:

Supplemental Video S1: Pretreated neutrophils.

Time-lapse imaging of resting neutrophils was performed using FV10i-LIV Laser Scanning Microscope (Olympus), Original maginification 600x, 1.8x zooming. 2.5 x 10^5 Neutrophils were plated on glass bottom dishes coated with 0.2% gelatin. Images and videos were analyzed by FV10i Fluoview Ver.3.0 software. Representative fields are presented from two independent experiments that in each experiment nine different fields were captured.

Supplemental Video S2: a2NTD treatment stimulates neutrophil polarization.

Time-lapse imaging of a2NTD treated neutrophils was performed using FV10i-LIV Laser Scanning Microscope (Olympus), Original maginification 600x, 1.8x zooming. 2.5 x 10^5 Neutrophils were plated on glass bottom dishes coated with 0.2% gelatin. Imaging of the neutrophils was acquired after treatment with 1 μg/ml of a2NTD. Representative fields are presented from two independent experiments that in each experiment nine different fields were captured. After a2NTD treatment, neutrophils started to adhere and refocusing of the microscope

lens was required, so the video is composed of three merged videos captured within thirty minutes of treatment.

Supplemental Video S3: Control neutrophils.

Time-lapse imaging of PBS (vehicle control) treated neutrophils was performed using FV10i-LIV Laser Scanning Microscope (Olympus), Original maginification 600x, 1.8x zooming. 2.5 x 10^5 Neutrophils were plated on glass bottom dishes coated with 0.2% gelatin. Representative fields are presented from two independent experiments that in each experiment nine different fields were captured. PBS treated neutrophils remained apolar (rounded).

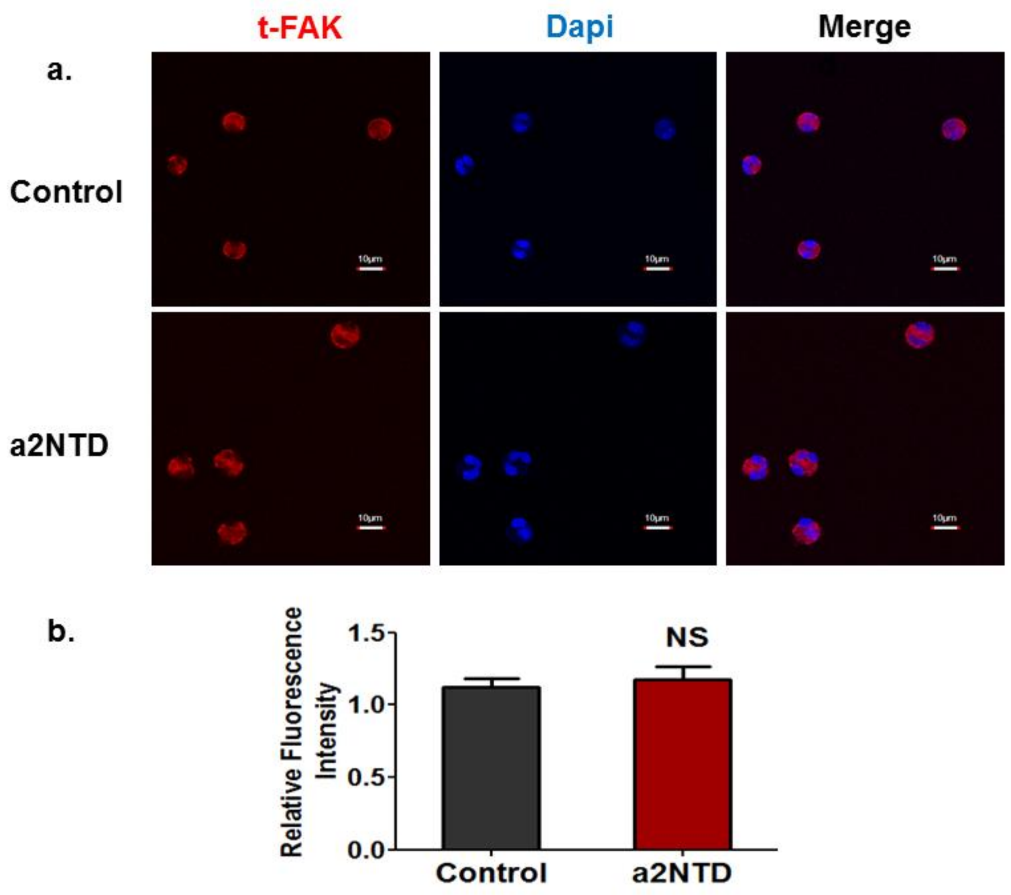

Supplemental Figure S4: Total FAK expression in a2NTD or vehicle control treated neutrophils.

a, Immunofluorescent staining of total FAK was performed in neutrophils. Freshly isolated neutrophils were plated in a poly Lysine coated 8 well chamber slides. After the incubation of cells with either PBS (control) or a2NTD for thirty minutes at 37 °C CO_2 incubator, cells were fixed, permeabilized, and stained with anti-FAK (red), Dapi (blue). Representative images were presented from 3 different experiments. b, Quatification of the intensity of the immunofluorescent staining of t-FAK in neutrophils was performed using FV10i Fluoview Ver.3.0 software. Data

are shown as relative average fluorescence intensity to control ± SEM. not statistically significant; NS, as compared with control neutrophils.

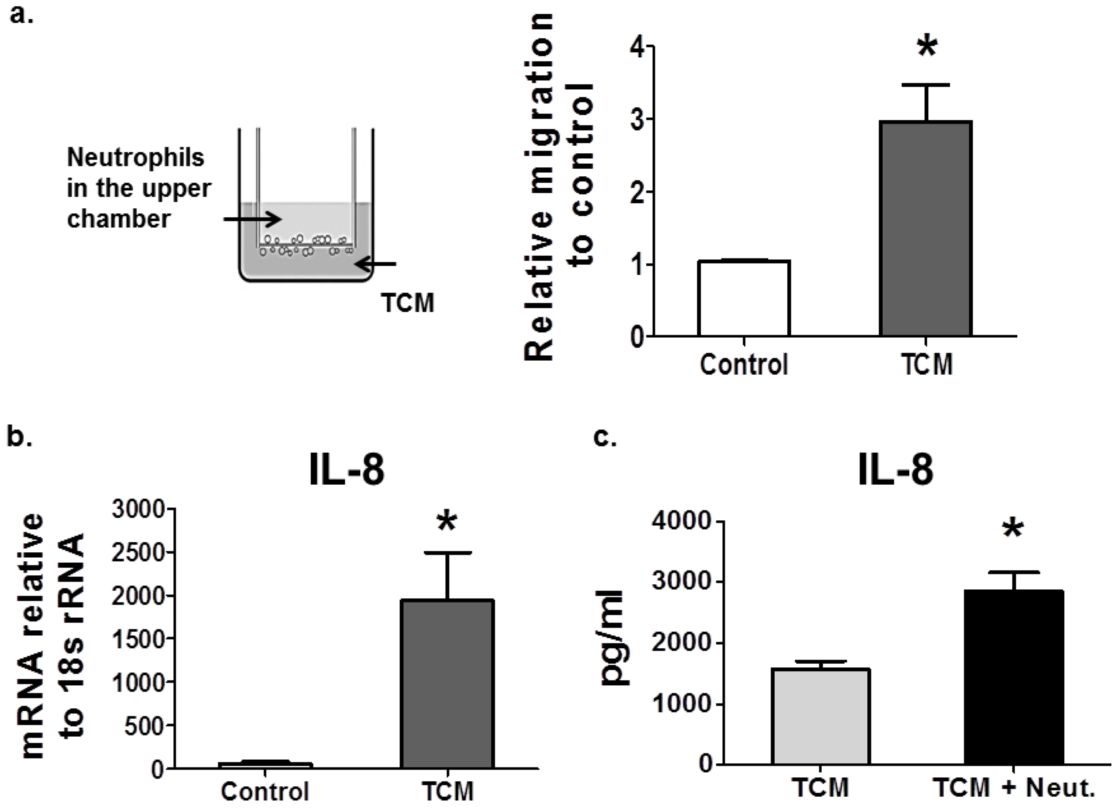

Supplemental Figure S5: Tumor condition media stimulates neutrophil migration and IL-8 secretion from neutrophils.

a, Trans-well migration assays were performed by plating 4 x 10^5 neutrophils into the upper chamber in serum free media, while either tumor condition media (TCM) collected from MDA MB-231 or 10% heat inactivated FBS complete media (control media) were placed in the bottom chamber. Cells were allowed to migrate for three hours at 37 °C CO_2 incubator. The migrated cells were quantified fluorometrically (n=3). The statistical significance was compared with

control neutrophils, **P <0.05.* b. Quantitative real time-PCR was performed to assess the mRNA gene expression of IL-8 in neutrophils cultured with TCM or control media for four hours at 37 °C in CO_2 incubator. Data were reported as mRNA expression relative to 18s rRNA ± SEM from three different experiments each was done in duplicate. **P <0.05,* as compared with control neutrophils. c. Quantitative analysis of IL-8 secreted levels was assessed using Luminex assay. IL-8 protein levels were determined in the supernatant collected from TCM treated neutrophils or in TCM after eighteen hours of treatment. Results presented as mean ± SEM from four different experiments. **P <0.05*, as compared with TCM.
